# Single-Point Periorbital Botulinum Neurotoxin Injection and Its Potential Role in Hematoma Risk Reduction: A Technical Report

**DOI:** 10.3390/toxins18080328

**Published:** 2026-07-28

**Authors:** Alexander Gardetto, Luis Thaler-Wolf

**Affiliations:** 1 Division of Plastic, Aesthetic and Reconstructive Surgery with Hand Surgery, Brixsana Private Clinic, Via Julius Durst 28, 39042 Bressanone, Italy; l.wolf@student.i-med.ac.at; 2 Clinic of Plastic, Reconstructive and Aesthetic Surgery, Padova University Hospital, Via Nicolo Giustiniani 2, 35128 Padova, Italy

**Keywords:** botulinum toxin type A, crow’s feet, lateral canthal rhytids, hematoma, injection technique, periorbital

## Abstract

Botulinum neurotoxin type A (BoNT-A) injection for lateral canthal rhytids is among the most frequently performed nonsurgical aesthetic procedures; however, procedure-related hematoma and ecchymosis remain frequent local adverse events associated with the multiple puncture sites required in the conventional three-point technique. This prospective single-center preliminary clinical observation evaluated a single-point intramuscular injection technique designed to reduce percutaneous vascular-layer penetrations while preserving treatment territory, dose, and intramuscular target localization. A total of 152 consecutive patients (104 female and 48 male) underwent bilateral treatment between May 2025 and May 2026 using a single skin entry point per side, positioned 10–15 mm caudal to and 10 mm temporal to the lateral canthus, followed by 18–20 mm intramuscular advancement within the lateral orbicularis oculi and continuous retrograde infusion of 12 U of onabotulinumtoxinA per side. Post-procedural assessment after injection demonstrated small unilateral ecchymosis in 2 of 304 treated sides (0.66%; Clopper–Pearson 95% Confidence Interval (CI), 0.08–2.36%). After the 10-day follow-up, no persistent bruising or other adverse events were reported, and all patients confirmed satisfaction with the treatment. At the structured one-year follow-up, the satisfactory reduction in dynamic lateral canthal rhytids and continued patient satisfaction with the treatment were confirmed, while a subset elected to return for a subsequent treatment session during the observation period. By reducing bilateral percutaneous venous-layer penetrations from six to two while maintaining dose and treatment-territory equivalence, the single-point intramuscular technique may represent a structurally coherent refinement of established BoNT-A treatment concepts for lateral canthal rhytids. Because this was an uncontrolled preliminary clinical observation, the observed rate should be interpreted as descriptive only; any potential reduction in hematoma risk remains hypothetical.

## 1. Introduction

**Figure 1:**
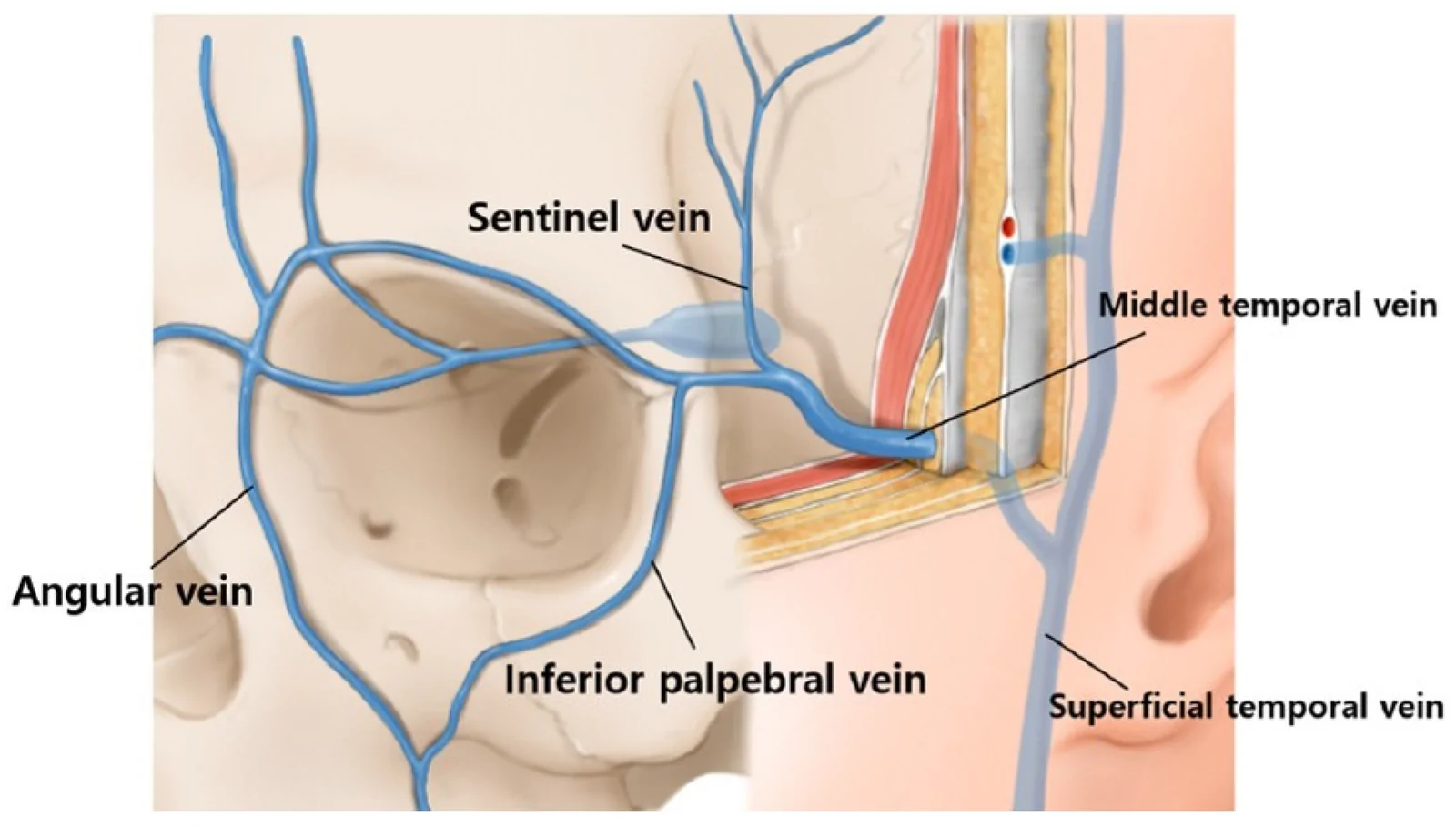
The veins of the periorbital regions. Reproduced from Yi et al., *Toxins* 2022, 14, 462 [7], under the Creative Commons CC BY 4.0 license.

Botulinum neurotoxin type A (BoNT-A) injection for lateral canthal rhytids (LCR), or crow’s feet lines, is among the most frequently performed nonsurgical aesthetic procedures worldwide [1,2]; onabotulinumtoxinA is approved for the treatment of LCR [2,3]. Despite the well-documented efficacy and safety profile of BoNT-A in this indication [4,5], procedure-related adverse events, particularly hematoma and ecchymosis, remain a persistent source of patient dissatisfaction. Most unwanted events are attributable to injection technique rather than to the toxin itself [6]. A thorough understanding of periorbital anatomy is essential for safe BoNT-A administration. The orbicularis oculi, the principal target for LCR treatment, encircles the eye and closes the eyelid; it comprises orbital, palpebral (preseptal and pretarsal), and lateral and medial banded portions [7]. The orbital portion, responsible for forceful eyelid closure and for generating lateral canthal rhytids during smiling, originates from the supraorbital and infraorbital margins and inserts into the canthal tendons and adjacent muscles. The mean linear distance from the lateral canthus to the lateral border of the muscle is 31 mm, and the lateral part is its thickest portion on ultrasonography. In the temporal region, the muscle lies superficially, within the layer extending to the superficial musculoaponeurotic system. The lateral periorbital wall is organized as discrete layers directly relevant to needle-based injections. From superficial to deep: skin; a thin subcutaneous layer containing the superficial periorbital venous plexus; the orbicularis oculi muscle; a sparse submuscular areolar plane; and the periosteum [7,8]. Here, the orbicularis muscle lies more superficially than the majority of facial muscles, separated from the dermis by only a thin subcutaneous stratum [8]. The medial zygomaticotemporal (sentinel) and inferior palpebral veins run within or just superficial to this subcutaneous layer and are therefore anatomically separated from the deeper orbicularis muscle belly that is the pharmacologic target of LCR treatment [7] (Figure 1).

The periorbital venous anatomy is of relevance to injection-related hematoma. The sentinel vein drains the superior and lateral orbital rim into the middle temporal vein before joining the superficial temporal vein; its piercing point lies at a mean of 26.8 ± 5.9 mm from the lateral canthus, within the range of conventional LCR injection sites [7]. The inferior palpebral vein drains the inferior palpebral and lateral orbicularis regions toward the angular vein near the inferior orbital rim. Most facial veins here are avalvular, permitting bidirectional flow. Taylor et al. demonstrated a plexus of large-caliber avalvular veins in the inner canthal and glabellar region, underscoring the vulnerability of periorbital vasculature to needle trauma [9].

The standard technique for LCR treatment uses three injection sites per side in the lateral orbicularis oculi, an approach originating from the early periocular guidelines of Matarasso S.L. and Matarasso A. [10]. This technique has since been refined in international consensus and review publications [2,11]. The first injection is placed at the level of the lateral canthus, 1.5 to 2.0 cm temporal to it and just temporal to the orbital rim; the second and third are placed 1.0 to 1.5 cm above and below the first at an approximate 30° medial angle [11]. Each injection delivers 0.1 mL containing 4 U of onabotulinumtoxinA, totaling 24 U (12 U per side). Lowe et al. identified this dose as most appropriate for crow’s feet in a dose–response trial [11,12]. An alternative pattern exists for LCR predominantly below the canthus [11]. Although this approach has shown consistent efficacy [5], each of the three sites per side requires an independent percutaneous puncture; therefore, a bilateral session entails six full-thickness needle passages through the skin and subcutaneous venous layer in immediate proximity to the avalvular periorbital plexus [11].

A critical, often underrecognized distinction exists between substance-related and procedure-related adverse events. In a meta-analysis of 32 randomized trials (9669 patients), Gostimir et al. found injection-site bruising, hematoma, or hemorrhage at nearly identical rates with BoNT-A and placebo (risk ratio 0.99; 95% confidence interval (CI) 0.66–1.49; *p* = 0.979), whereas events attributable to the toxin’s pharmacologic action, such as eyelid or eyebrow malposition (risk ratio 3.55; *p* < 0.001) and headache (risk ratio 1.45; *p* = 0.003), were significantly more frequent with BoNT-A [4]. An earlier meta-analysis by Brin et al. (1678 participants) likewise confined significant BoNT-A versus placebo differences to substance-related events [13]. Consistently, a randomized crow’s feet trial reported mild bruising in 11–25% of patients, comparable to placebo [14]. Together, these data indicate that hematoma and bruising are predominantly procedure-related, driven by mechanical vascular trauma rather than the neurotoxin [4,13,14]; bruising is the most prevalent adverse effect of BoNT injection in the lateral canthal region [7].

Cadaveric studies show that repeated penetrations progressively increase skin penetration force, with 30-gauge needles exhibiting a significant 30% rise by the fifth insertion (*p* = 0.007) from cumulative tip deformation [15]. Each additional puncture, therefore, raises both the probability of vascular injury and the tissue trauma from increasing tip resistance. Despite the enormous global volume of BoNT-A procedures—over 200,000 treatments in a single multicenter cohort, including a subset of 37,955 (19%) LCR treatments—the focus of this work and similar ones lies on the most unwanted events [16]. Overall, analysis of 10,577 reports comprising 29,471 adverse events related to BoNT-A treatment identified injection-site reactions such as pain (9.3%) and swelling (6.4%) as the most frequently reported [17]. The literature lacks a systematic approach to reducing procedure-related hematoma by modifying the injection technique, as well as complication incidence rates for treatment with BoNT-A in the periorbital region. Current evidence focuses overwhelmingly on substance-related efficacy and safety, while the mechanical determinants of bruising receive comparatively little attention.

We present a single-point injection technique for periorbital BoNT-A that uses the anatomical separation between the subcutaneous venous network and the underlying orbicularis oculi muscle to distribute the toxin along a single intramuscular tract across the established three-point target zone. By reducing the number of needle passages, this technique may lower the risk of procedure-related hematoma while maintaining comparable toxin distribution.

## 2. Results

The observation cohort comprised 152 consecutive patients, corresponding to 304 treated periorbital sides, with a mean age of 49 ± 11 years and a female predominance of 68.4%. Demographic and injury-related data are summarized in Table 1.

### 2.1. Primary Outcome: Procedure-Related Hematoma and Ecchymosis

At immediate post-procedural assessment, a small ecchymosis was observed in 2 of 304 injected sides (0.66%). Each was unilateral, occurring in 2 separate patients (2 of 152; 1.3%). In both cases, the ecchymosis was confined to the immediate periorbital treatment zone and required no additional intervention. No hematoma, delayed manifestation, secondary bleeding, or extension of discoloration beyond the treatment zone was reported.

A punctate petechia at the skin entry, observed on a subset of sides as an expected consequence of percutaneous injection, was not classified as a procedure-related hematoma and was not counted toward the primary outcome. Although the observed per-side proportion was low at 0.66%, the corresponding Clopper–Pearson 95% CI was relatively wide (0.08–2.36%), reflecting the limited precision imposed by the small number of observed events. Both procedure-related events occurred in patients with a documented history of prior periorbital BoNT-A treatment, with no procedure-related adverse event recorded at those prior sessions.

### 2.2. Ten-Day Follow-Up

All 152 patients (100%) were successfully contacted at the structured 10 (+2)-day telephone follow-up. No patient (0%; 0 of 304 sides) reported persistent bruising, swelling, or any other adverse event. The 2 patients with a small ecchymosis at immediate assessment reported no residual bruising or discoloration at the 10-day contact, consistent with complete resolution by that timepoint.

### 2.3. Patient Satisfaction

All 152 patients responded affirmatively to the structured satisfaction question (“Were you satisfied with the treatment?”) at the follow-up call. This single question was recorded as a pragmatic satisfaction check within the safety-oriented follow-up, not as a validated efficacy measure, and should not be read as evidence of aesthetic efficacy.

### 2.4. Other Adverse Events

No eyelid or eyebrow malposition, dry-eye symptoms, headache, facial-expression asymmetry, hypersensitivity reaction, or other adverse event attributable to either the injection procedure or the pharmacologic action of onabotulinumtoxinA was observed by the treating physician at immediate assessment or reported by any patient at the 10 (+2)-day telephone follow-up.

### 2.5. Clinical Effect at End-of-Observation Follow-Up

As an exploratory, uncontrolled observation without validated outcome measures, a structured follow-up was conducted at the end of the one-year observation period. These data are reported solely to document the feasibility and real-world context of the technique and carry no inferential weight regarding efficacy. Of the 152 patients included in the study, 131 (86.2%) participated in this follow-up. Among these patients, a satisfactory reduction in dynamic lateral canthal rhytids was recorded in 127 (97%), and all 131 patients (100%) reported satisfaction with the treatment outcome. No patient required a corrective top-up injection because of an inadequate clinical effect during the observation period. A retrospective review of appointment records identified 89 of 152 patients (58.5%) who elected to continue treatment during the observation period, with a median interval of 5.1 months. Because efficacy was not prospectively assessed using validated wrinkle-severity scales or standardized photography, and no comparator arm was included, these observations cannot be interpreted as evidence of comparative or absolute treatment efficacy. All findings are summarized in Table 2.

## 3. Discussion

The present preliminary clinical observation describes a single-point intramuscular injection technique for BoNT-A treatment of crow’s feet lines that reduces the number of percutaneous vascular-layer transits while preserving the established pharmacologic and anatomical principles of the conventional three-point approach. This technique is grounded in the layered anatomy of the lateral periorbital region. The venous structures, including the sentinel and inferior palpebral veins, are located within or immediately superficial to the subcutaneous tissue, whereas the orbicularis oculi muscle belly occupies a distinct deeper plane adjacent to the periosteum [7,8]. By selecting a single vein-free skin entry point and subsequently advancing the needle within the muscle plane, the venous plexus is traversed only once per side. In contrast, the standard three-point technique requires multiple passages through the vascular layer [11]. This reduction in percutaneous vascular transits provides a plausible anatomical rationale for the ecchymosis rate (0.66%; 95% CI 0.08–2.36%) observed in the present cohort. However, without a comparator, this single-arm observation cannot link the technique to the ecchymosis rate and remains hypothesis-generating rather than evidence of superiority.

An additional consideration relates to progressive needle-tip deformation during repeated skin penetrations. Experimental work in cadaveric skin by Tansatit et al. demonstrated that repeated insertions of 30-gauge needles progressively blunt the bevel, resulting in an approximately 30% increase in penetration force by the fifth insertion (*p* = 0.007), which may contribute to greater soft-tissue trauma [15]. Reducing bilateral skin penetrations from six to two may therefore limit not only direct vascular trauma but also the progressive mechanical tissue disruption associated with repeated needle use. Although penetration force and pain perception were not directly measured in the current observation, the absence of patient-reported adverse events during follow-up is compatible with this hypothesis. Furthermore, hemorrhagic side effects of periocular BoNT-A do not appear to be increased in patients on antithrombotic therapy [18], and hematoma frequency was only marginally elevated under oral anticoagulation (3.1% vs. 1.8%) [19], supporting a mechanical, procedure-related mechanism.

The proposed approach also differs conceptually from recently advocated superficial injection strategies. Several authors have recommended intradermal or subdermal placement of BoNT-A in order to avoid the superficial venous plexus and rely on passive diffusion of the toxin into the orbicularis oculi muscle [7,8]. In contrast, the single-point technique instead places the needle deep to the plexus within the orbicularis muscle belly, the same anatomical and pharmacologic target described by the international consensus three-point recommendations [2,11], delivering toxin within the muscle tissue rather than relying on diffusion from a superficial depot. The intramuscular target was described by Vachiramon et al. in a split-face controlled trial of 48 patients comparing a three-point intramuscular technique with a six-point intradermal technique using equivalent doses of incobotulinumtoxinA for crow’s feet. A significantly greater reduction in periorbital wrinkle indentation index was reported on the intramuscular side at 8, 12, and 16 weeks, together with a significantly longer time to clinical relapse [20]. Intramuscular delivery into the orbicularis oculi has been associated with at least comparable periorbital efficacy and duration relative to intradermal placement, supporting the anatomical and pharmacologic rationale for targeting the muscle plane. In the present technique, this intramuscular approach is combined with a reduction in percutaneous venous-layer traversals through the use of a single-entry, retrograde distribution pattern. However, the term “equivalence” in this observation refers strictly to dose, pharmacologic exposure, and cranio-caudal treatment territory in relation to the conventional three-point technique. Aesthetic effectiveness was not quantitatively assessed, and no equivalence in clinical aesthetic outcome can therefore be inferred. The findings should also be interpreted in the context of the limited existing literature on linear injection techniques in the periorbital region. Li et al. described a related retrograde linear injection technique [21], a split-face randomized controlled trial (RCT) of 28 patients that found a transient 1-week wrinkle-improvement disadvantage for linear versus spot injection in the periorbital region (median 25.9% vs. 48.1%; *p* < 0.03), with no significant difference at 1, 3, or 6 months. However, that study differed from the present approach with regard to both injection depth and toxin dose, as injections were performed subcutaneously and with a lower total dose per side (6 vs. 12 U per side) [11,21]. These differences in depth and dose preclude a direct comparison with the present technique. The one-year observational follow-up data, collected without a comparator arm or validated outcome measures, are reported as descriptive context only and are not intended to support conclusions regarding the clinical efficacy of the technique; the observed return rate is a favorable but uncontrolled signal.

Several limitations must be acknowledged. This was a single-center, prospective, preliminary clinical observation performed by a single experienced surgeon. The reported outcomes reflect observational rates within a consecutive cohort rather than a direct comparison against the conventional three-point technique. Furthermore, the outcomes may partly reflect the operator’s experience in periorbital injection anatomy and technique, potentially limiting generalizability to less experienced injectors. The immediate post-procedural assessment was performed by the same treating physician who performed the injection, without an independent blinded observer; this constitutes an unblinded observer effect on the primary endpoint and could plausibly bias the classification of borderline punctate petechia versus small ecchymosis. No validated wrinkle-severity scale was applied; the primary endpoint relied on immediate physician documentation and subsequent patient self-report. The temporal evolution of the two ecchymoses was captured at two discrete timepoints only (immediate assessment and the 10 (+2)-day telephone contact), so a continuous reconstruction of appearance and resolution between these timepoints could not be performed. Patients on chronic antiplatelet or anticoagulant therapy were not included; procedural safety in these subgroups, therefore, lies outside the scope of the present observation.

## 4. Conclusions

The single-point intramuscular technique may represent a structurally coherent refinement of established BoNT-A treatment concepts for crow’s feet lines by reducing vascular-layer penetrations while maintaining identical treatment territory, dose, and intramuscular target localization, thereby providing a plausible mechanistic basis for a potential reduction in procedure-related bruising, with the aim of preserving therapeutic intent. Because the study was uncontrolled, the observed per-side ecchymosis rate of 0.66% (Clopper–Pearson 95% CI, 0.08–2.36%) should be interpreted descriptively and does not demonstrate superiority over the conventional technique. A prospective split-face study using validated wrinkle-severity scales and directly comparing the single-point and conventional three-point techniques is therefore required before definitive conclusions can be drawn regarding their relative clinical efficacy.

## 5. Materials and Methods

This single-center, prospective case series describes the development, standardization, and clinical application of a single-point injection technique for BoNT-A treatment of LCR, conducted in one private aesthetic surgery practice at Brixsana Private Clinic (Julius Durststreet 28, 39042 Bressanone, Italy) between May 2025 and May 2026. The primary aim was to present a technical refinement of an established, pharmacologically validated treatment, supplemented by observational documentation of procedure-related complications and patient satisfaction in a consecutive cohort. No comparative arm was included; the standard three-point technique is referenced solely for dose equivalence and is not analyzed as a control. The absence of a comparator group is acknowledged as a limitation in the Section 3.

### 5.1. Patient Population

A consecutive series of 152 patients (104 female and 48 male) presenting for aesthetic treatment of LCR during the observation period was enrolled. All received bilateral periorbital treatment using the single-point technique, representing 304 injected sides in total. The inclusion criterion was the presence of dynamic or mixed dynamic-static LCR for which the patient sought BoNT-A treatment. Exclusion criteria comprised the standard contraindications to BoNT-A therapy, namely known hypersensitivity to the active substance or excipients, active cutaneous infection at the injection site, pregnancy or lactation, neuromuscular junction disorders, and concomitant aminoglycoside therapy, together with chronic antiplatelet or anticoagulant therapy. Patients with prior periorbital BoNT-A treatment were not excluded. All patients provided written informed consent for treatment and for the use of anonymized clinical data for educational and scientific purposes.

### 5.2. Anatomical Considerations

The technique is grounded in the topographic relationships of the lateral orbicularis oculi muscle and the superficial periorbital venous plexus, as detailed in the Introduction (Figure 1). The lateral canthus serves as the reference for the caudal entry, which is placed 10–15 mm caudally and 10 mm temporally to it, positioning the needle on a vertical line that permits cranial advancement parallel to the lateral orbital rim. The sentinel and inferior palpebral veins are mapped visually before injection; the sentinel piercing point lies at a mean of 26.8 ± 5.9 mm from the lateral canthus, within the planned treatment zone [7]. The orbicularis muscle plane, superficial to the periosteum and deep to the subcutaneous venous plexus, is the target compartment. This plane separation is the central principle of the technique: the venous network is traversed only at the single skin entry, after which the needle advances within the muscle, beneath the vascular plane, and above the periosteum.

### 5.3. Materials

OnabotulinumtoxinA was used in all patients. Each 100 U vial was reconstituted with 2.5 mL of preservative-free 0.9% sodium chloride solution, yielding a final concentration of 4 U per 0.1 mL. The reconstituted toxin was drawn into a 1 mL insulin syringe fitted with a 30-gauge, 20 mm hypodermic needle. Skin antisepsis was performed with an alcohol-based disinfectant. One needle was used for both sides in each patient. Whereas the conventional three-point technique typically reuses the same needle across up to six skin penetrations per treatment, the present protocol reduces this to two skin penetrations. The procedure is performed with the patient semi-reclined under standard procedure-room illumination. Each side is treated sequentially according to the following five standardized steps.

### 5.4. Technique Description

**Figure 2:**
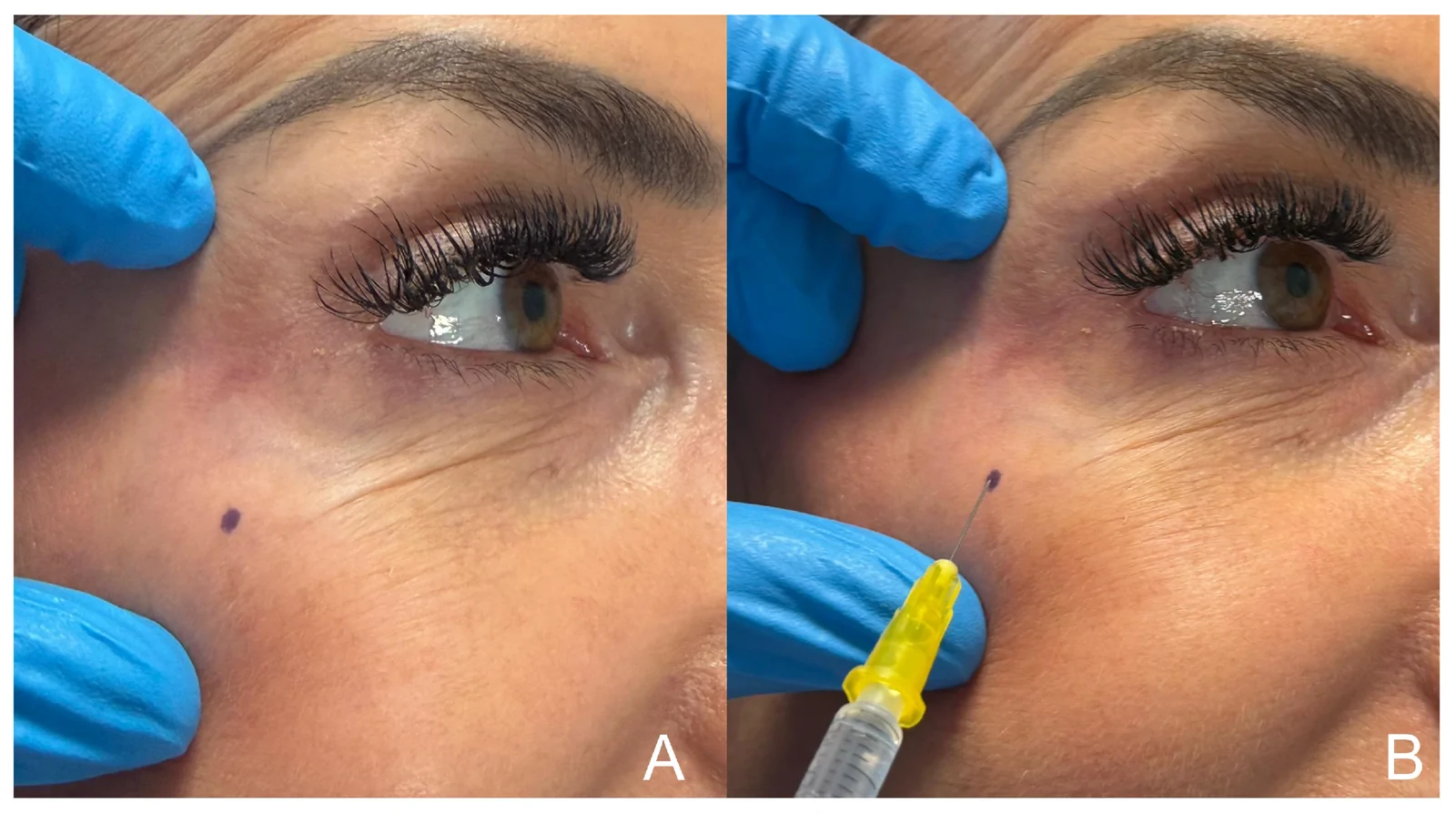
(**A**) Visual identification of superficial periorbital veins and selection of a vein-free entry point. (**B**) Needle entry at the caudal-temporal insertion point into the orbicularis oculi muscle plane.

*Step 1. Skin Tension.* The non-dominant hand places the lateral periorbital skin under gentle caudal traction. This stabilizes the soft-tissue envelope and reduces patient-perceived discomfort at needle entry. (Figure 2A).

*Step 2. Vein Identification.* The superficial periorbital venous plexus is identified visually with the naked eye, aided by skin tension and, when needed, oblique illumination. If a visible vein lies at the planned entry, the entry is shifted a few millimeters within the defined caudal-temporal landmark range to a vein-free location. Vessels overlying the trajectory above the entry require no evasive maneuvers, since advancement occurs within the muscle belly, deep to the subcutaneous venous network. (Figure 2A).

*Step 3. Needle Entry.* The needle is introduced bevel up at the defined caudal entry point, 10–15 mm caudal to and 10 mm temporal to the lateral canthus, traversing the skin and subcutaneous tissue at the vein-free location identified in Step 2. It is then placed within the muscle belly of the lateral orbicularis oculi, superficial to the periosteum and deep to the subcutaneous venous plexus. Depth is confirmed by tactile feedback; bony contact is avoided (Figure 2B).

**Figure 3:**
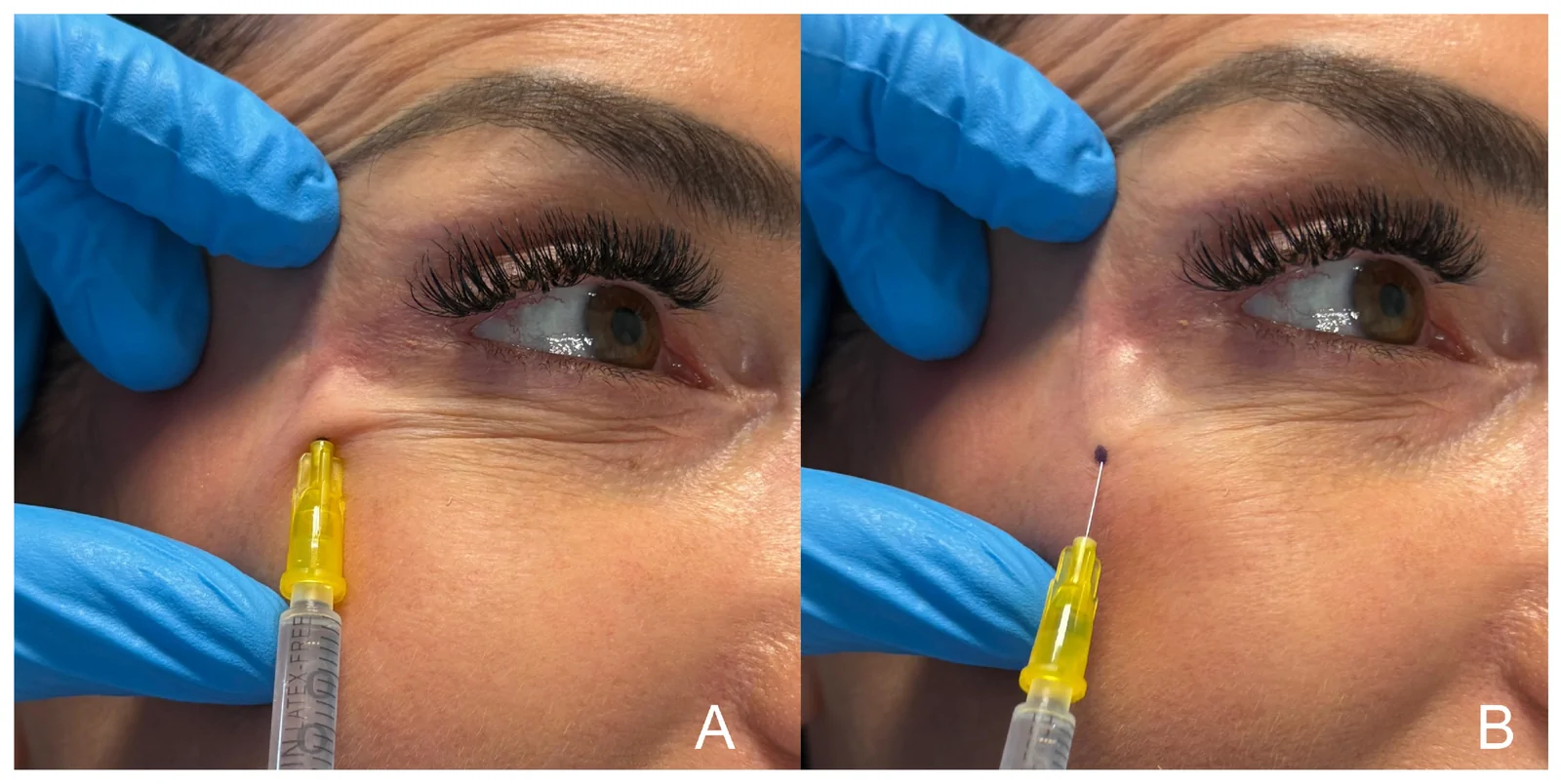
(**A**) Cranial intramuscular needle advancement parallel to the lateral orbital rim. (**B**) Continuous retrograde BoNT-A injection during slow needle withdrawal.

*Step 4. Cranial Advancement.* Within the orbicularis muscle belly, beneath the venous plexus and superficial to the periosteum, the needle is advanced cranially along a straight vertical trajectory parallel to the lateral orbital rim. Total intramuscular advancement is about 18–20 mm, terminating at the superior aspect of the treatment zone (Figure 3A).

*Step 5. Controlled Withdrawal and Continuous Distribution.* BoNT-A is delivered as a continuous linear retrograde infusion: the plunger is depressed at a steady rate while the needle is slowly withdrawn along the original tract, distributing the toxin evenly across the full vertical extent of the treatment zone. A total volume of 0.3 mL (12 U) is delivered per side, and the same needle is then used for the contralateral injection (Figure 3B) and (Video S1).

### 5.5. Dosing Protocol

The total dose was 12 U of onabotulinumtoxinA in 0.3 mL per side, for a bilateral total of 24 U in 0.6 mL. This is identical to the international consensus for the three-point technique, in which 4 U is delivered at three sites per side. Lowe et al. identified 12 U per side in a randomized trial as the most appropriate dose for crow’s feet [11,12]. The single-point technique, therefore, modifies the mode of toxin delivery, leaving total pharmacologic exposure unchanged and enabling a direct dose-equivalent reference to the established standard.

### 5.6. Outcome Measures and Follow-Up

The primary outcome was the incidence of procedure-related ecchymosis, analyzed per side at the index treatment (denominator 304) because each side constitutes an independent procedural event. Two structured assessments were performed. First, an immediate post-procedural assessment by the physician recorded whether any visible hematoma, ecchymosis, or other acute adverse event in the treatment zone occurred. A small ecchymosis was defined as a discrete area of visible discoloration > 1 mm, extending beyond the needle insertion point. A minor punctate petechia, defined as a pinpoint hemorrhagic spot ≤ 1 mm confined to the needle insertion site itself, was considered an inevitable consequence of percutaneous injection and was not classified as a hematoma. Subsequently, a structured telephone follow-up at 10 (+2) days asked whether any bruising, swelling, or other adverse event had been noticed since the procedure, with the side of occurrence recorded. The follow-up window was chosen to match the natural history of procedure-related ecchymosis; it is not intended as an aesthetic efficacy timepoint.

Patient satisfaction was initially assessed at the patient level (denominator: 152) during the 10-day telephone follow-up using a single dichotomous question: “Were you satisfied with the treatment?” A further structured follow-up was conducted at the end of the one-year observation period to obtain supportive information on the clinical effect. At this follow-up, three patient-level outcomes were documented: whether a satisfactory reduction in dynamic lateral canthal rhytids had been achieved, whether the patient was satisfied with the treatment outcome, and whether a corrective top-up injection had been required because of an inadequate effect. In addition, repeat treatments performed during the observation period were identified through a retrospective review of the appointment records. These outcomes were based on clinical assessment, patient reports, and treatment records. No validated quantitative scale or standardized photographic assessment of wrinkle improvement was used. Accordingly, these findings are presented as descriptive, supportive observations and not as a controlled assessment of efficacy. A prospectively designed comparative study using validated outcome measures would be required to formally evaluate the clinical efficacy of the technique.

### 5.7. Statistical Analysis

No inferential statistical testing was performed. Results are reported as absolute numbers and percentages: per side for the primary outcome (denominator 304) and per patient for satisfaction (denominator 152). Because only two events occurred, an exact 95% CI using the Clopper–Pearson method was also calculated for the primary outcome, reflecting the uncertainty associated with an event rate based on so few observations.

## Figures and Tables

**Table 1:** Baseline demographic characteristics and prior BoNT-A history of the observation cohort (*n* = 152). Standard deviation (SD).

Characteristic	Value
Total patients, *n*	152
Injected sides, *n*	304
Female sex, *n* (%)	104 (68.4)
Male sex, *n* (%)	48 (31.6)
Mean age ± SD, years	49 ± 11
Age range, years	23–76
Prior periorbital BoNT-A treatment, *n* (%)	117 (77)
BoNT-A-first treatment, *n* (%)	35 (23)
Procedure-related adverse events in prior BoNT-A sessions, *n* (%) *	8 (6.8)

* Adverse events recorded in the medical history of patients with a documented history of prior periorbital BoNT-A treatment sessions (elsewhere or in this practice).

**Table 2:** Clinical effect and follow-up outcomes at the end of the one-year observation period.

Outcome	Value
Participants included in the end-of-observation follow-up, *n* (%)	131 (86.2)
Satisfactory reduction of dynamic lateral canthal rhytids, *n* (%) *	127 (97)
Patient satisfied with treatment outcome, *n* (%) *	131 (100)
Corrective top-up injection for inadequate effect, *n* (%)	0 (0)
Returned for another treatment during observation, *n* (%)	89 (58.5)
Median interval to next treatment, months	5.1

* Based on subjective patient self-report.

## Data Availability

The original contributions presented in this study are included in the article/Supplementary Materials. Further inquiries can be directed to the corresponding author.

## Supplementary Materials

The following supporting information can be downloaded at https://www.mdpi.com/article/10.3390/toxins18080328/s1, Video S1: Detailed demonstration of the injection technique.

## Abbreviations

The following abbreviations are used in this manuscript:

BoNT-A	Botulinum neurotoxin type A
LCR	Lateral canthal rhytids
RCT	Randomized controlled trial
CI	Confidence interval
